# Characterization of the spectrum of Korean inflammatory demyelinating diseases according to the diagnostic criteria and AQP4-Ab status

**DOI:** 10.1186/1471-2377-14-93

**Published:** 2014-04-29

**Authors:** Sung-Min Kim, Patrick Waters, Mark Woodhall, Ji Won Yang, Hyeran Yang, Jee-Eun Kim, Jung-Joon Sung, Kyung Seok Park, Kwang-Woo Lee

**Affiliations:** 1 Department of Neurology, Seoul National University, College of Medicine, Seoul, Korea; 2 Nuffield Department of Clinical Neurosciences, Neuroimmunology group, Oxford, UK; 3 Department of Neurology, Seoul National University Bundang Hospital, 166 Gumi-Ro, Bundang-Gu, Seongnam-Si, Gyeonggi, Korea; 4 Department of Neurology, Seoul Medical Center, Seoul, Korea

**Keywords:** Neuromyelitis optica, Multiple sclerosis, Anti-aquaporin-4 antibody, Idiopathic inflammatory demyelinating disease of the central nervous system, Korean

## Abstract

**Background:**

The relative frequencies of demyelinating diseases among Korean patients with idiopathic inflammatory demyelinating disease of the central nervous system (IIDD) have not been sufficiently studied. We therefore describe a cohort of 203 patients with IIDD from three centers in Korea whose syndromes were identified precisely according to international clinical criteria and autoantibody to aquaporin 4 (AQP4-Ab) status.

**Methods:**

In total, 260 consecutive patients were screened and 203 were included from three hospitals in Korea. All were tested for AQP4-Ab by using a cell-based assay. Patients who met the criteria for definite neuromyelitis optica (NMO) or had a positive AQP4-Ab test result were defined as the NMO group. Among the others, patients were assessed if they had acute disseminated encephalomyelitis, multiple sclerosis (MS), acute transverse myelitis, optic neuritis, or other demyelinating disease as a clinically isolated syndrome of the brain.

**Results:**

Eighteen percent of patients were classified as the NMO group, 2% as acute disseminated encephalomyelitis, 18% as MS, 41% as acute transverse myelitis, 11% as optic neuritis, and 8% as other clinically isolated syndrome of the brain. AQP4-Ab was positive in 18% of patients and the relative frequency of NMO to MS (NMO/MS ratio) was 1.06. The mean duration of follow up in our patients was 64 months.

**Conclusions:**

Among Korean patients with idiopathic inflammatory demyelinating diseases, the incidence of NMO may be similar to that of MS, and the overall positivity of AQP4-Ab could be lower than previously reported. In addition, acute transverse myelitis that is not associated with MS or NMO can be relatively common in these patients. Further population-based studies with AQP4-Ab are needed to determine the exact incidence of NMO and other idiopathic inflammatory demyelinating diseases in Korea.

## Background

Idiopathic inflammatory demyelinating disease of the central nervous system (IIDD) refers to a wide spectrum of disease entities that mostly consist of multiple sclerosis (MS) [1], neuromyelitis optica (NMO) [2,3], acute disseminated encephalomyelitis (ADEM) [4], acute transverse myelitis (ATM) [5], and optic neuritis (ON) [6].

NMO is distinguished from MS by the presence in the serum of a pathogenic autoantibody to aquaporin-4 (AQP4-Ab) [7], by severe optic and spinal attacks [8], and by the presence of a severely disrupted blood–brain barrier [9]. The relative frequency of NMO to that of MS (NMO/MS ratio) was previously reported to be high in Thailand (1.4) [10] and Japan (0.29–0.59) [11,12], compared to that in Europe (0.024) [13] and Latin America (0.073–0.26) [14].

However, the NMO/MS ratio, as well as the relative frequencies of other demyelinating diseases such as ADEM, ATM, and ON among Korean patients with IIDD, have not been sufficiently studied.

The aim of this study was to describe a cohort of 203 patients from three centers in Korea with IIDD of the central nervous system, using international clinical and serological criteria.

## Methods

### Patients

In total, 260 consecutive patients who were suspected as having IIDDs such as definite NMO, NMO spectrum disorder (NMOSD) [2,3], ADEM [4], MS [1], ATM [5], ON [6], or a clinically isolated syndrome (CIS) of the brain [15] and whose serum was tested at the John Radcliffe Hospital, Oxford, were screened [16].

All provided written consent and visited Seoul National University Hospital or Seoul National University Bundang Hospital between September 1, 2009, and June 30, 2012, or Seoul Medical Center between March 1, 2011, and June 30, 2012. Excluded were patients who had incomplete medical records (*n* = 3), no magnetic resonance imaging (MRI) data (*n* = 2), were diagnosed with diseases other than IIDDs (such as infectious, vascular, tumorous, degenerative, or metabolic conditions; *n* = 48), were referred from a foreign hospital (*n* = 1), or were followed for less than 6 months (n = 3). In total, 203 patients were finally included in the study; the duration of their follow-up was 64.42 ± 60.03 months (mean ± standard deviation).

### Classification of patients

We evaluated the diagnoses of patients using the following steps:

Step 1: Identification of patients who met the revised diagnostic criteria for definite NMO [2].

Step 2: Patients who did not meet the diagnostic criteria for NMO [2] were dichotomized according to their test results for AQP4-Ab. Those with positive test results were included in the NMO group, and were assessed if they had the clinical features of NMOSD [3]. These features included 1) longitudinally extensive myelitis involving three or more vertebral segments, 2) ON with recurrent or simultaneous bilateral events, and 3) ON or myelitis associated with symptomatic brain lesions typical of NMO [3]. Consistent with recent recommendations [1], the criteria for opticospinal MS [11] were not included.

Step 3: Assessment of patients to determine whether they met the proposed criteria for ADEM [4], among those who were found not to have AQP4-Ab in the above step.

Step 4: Assessment of patients who did not fulfill the above criteria, using the 2010 international panel diagnostic criteria for MS [1].

Step 5: Assessment of patients who did not meet any of the above criteria to determine whether they had a clinically isolated syndrome of the brain [17], ATM [18-20], or ON [6].

### AQP4-Ab testing

Sera were taken, immediately centrifuged, and stored at −80°C, according to standard protocols [21]. They were tested for the presence of AQP4-Ab at the John Radcliffe Hospital, Oxford, UK using a cell-based assay (CBA) with recombinant AQP4, as described previously [22,23].

### Standard protocol approval, registration, and patient consent

This study was approved by the Seoul National University Bundang Hospital (IRB number: B-1007-105-401) and Seoul National University Hospital Institutional Review Board (IRB number: H-1012-023-317). All patients provided written informed consent before participating.

## Results

### Number of patients and AQP4-Ab–positive cases in the individual disease groups

Of the 203 patients with IIDDs, 17 met the revised diagnostic criteria for definite NMO [2] and 16 (94%) of these patients had positive AQP4-Ab test results [2]. Twenty-one patients who did not meet the NMO criteria also had positive AQP4-Ab test results, nineteen of whom showed clinical features of NMOSD. Two patients with positive AQP4-Ab test results did not have the clinical features of NMOSD. One of these patients had symptoms of diplopia and an isolated brain lesion involving the AQP4-rich periaqueductal area [24], and the other had recurrent mild symptoms and signs of myelitis (e.g. hypesthesia, pain in lower extremities, voiding difficulty, hyper-reflexive deep tendon reflex) but no definite lesion that could be seen on repeated spinal MRI.

Of the 165 patients who did not meet the revised diagnostic criteria for definite NMO [2] and did not have AQP4-Ab, 3 met the proposed criteria for ADEM [4] and 36 met the International Panel criteria for MS [1]. All of our patients with ADEM were monophasic with a mean follow up duration of 26.8 months, and had no oligoclonal band.

Of the remaining patients, 19 had clinically isolated syndrome of the brain, 84 had ATM, and 23 had ON. The ATM cases without evidence of either NMO or MS consisted of 50 patients with longitudinally extensive transverse myelitis (LETM) and 34 patients with acute partial transverse myelitis (APTM). The mean age at onset was 44.73 ± 13.90 y, 64 patients (76%) were male, and the follow up duration was 45.95 ± 38.73 months. The relative incidence of these patients was high, being 41.4% (84 out of 203) of our cohort.

**Figure 1:**
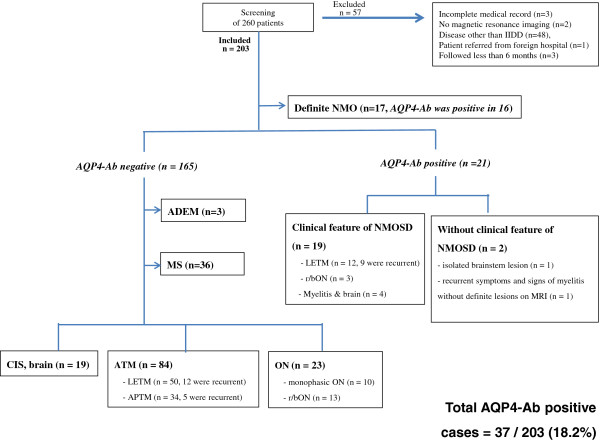
**Diagnostic flow and AQP4**-**Ab positivity in individual groups.** Abbreviations: ADEM = acute disseminated encephalomyelitis, APTM = acute partial transverse myelitis, AQP-Ab = aquaporin-4 autoantibody, CIS, brain = clinically isolated syndrome of the brain, IIDDs = idiopathic inflammatory demyelinating diseases of the central nervous system, LETM = longitudinally extensive transverse myelitis, MRI = magnetic resonance imaging, MS = multiple sclerosis, myelitis and brain = myelitis associated with symptomatic brain lesions typical of NMO, n = number, NMO = neuromyelitis optica, NMOSD = neuromyelitis-optica spectrum disorder, ON = optic neuritis, r/blON = recurrent or bilaterally simultaneous optic neuritis.

The overall incidence of cell-based anti-AQP4-Ab positivity [25] was 18.2% in our Korean cohort with IIDDs (Figure 1).

### Ratio of NMO to MS in Korean IIDD

The number of patients in our NMO group was 38, including 17 patients with definite NMO and 21 AQP4-Ab–positive patients with or without the clinical features of NMOSD. Therefore, the NMO/MS ratio was 1.06 (38/36) in our cohort (Figure 1).

## Discussion

The present study revealed the following findings in 203 Korean patients with IIDDs from three major hospitals. (1) In our cohort, the NMO/MS ratio was 1.06, which implies that the number of patients with NMO was almost the same as the number with MS. (2) The AQP4-Ab test was positive in 18.2% of Korean patients with IIDDs. (3) ATM not associated with MS or NMO was common in Korean patients with IIDD and accounted for 41% of these patients.

The NMO/MS ratio was 1.06 in our Korean cohort and was higher than that in a Danish population (0.024) [26] and in a Japanese study (0.29–0.59) [11,12]. Combining our figure for the ratio with the prevalence of MS in Korea (3.5–3.6 cases per 100,000 individuals) [27] may enable us to estimate the crude prevalence of NMO in Korea. However, additional population-based studies are needed to determine the exact values.

As expected, the overall AQP4-Ab positivity among our Korean patients with IIDDs (18.2%) was higher than that among Caucasian populations (1.7–7.2%) [26,28], which may be due to the higher incidence of MS in Caucasian patients with IIDDs (50.8–80%) [26,28] compared to that in our sample (17.7%). However, our AQP4-Ab positivity rate was much lower than the previously reported high AQP4 positivity rate in single-center studies in Thailand (39.3%) [10] and Korea (33%) [29], which could have been associated with selection bias in the single-center designs of those studies [25].

In our cohort, the incidence of ATM patients without evidence of NMO or MS was high (43.8%). The exact cause of this phenomenon is not clear. However, it could be related to one or more of the following: First, the prevalence of MS was reported to be low (3.5–3.6/100,000) in Korea [27], compared to that in Western countries (>150/100,000 in northern Europe) [30]. This makes the incidence of MS among Korean patients with IIDD relatively low, which in turn, could have brought about the relatively high incidence of other IIDDs, such as ATM, in our cohort. Second, although we have adopted a highly sensitive assay method for AQP4-Ab that uses recombinant AQP4 antigen, a recent study has shown that using a combination of different assays and/or retesting can improve the sensitivity of the AQP4-Ab test in a small number of patients [31]. Therefore, we speculate that a minor portion of our patients with ATM could have been more accurately diagnosed as having NMO with the use of combination assay methods or serial retesting. Third, the observed difference in ATM incidence could have been due to a difference in genetic susceptibility between Asians and Caucasians [32]. Fourth, some of these patients with ATM might have been diagnosed as having MS with a longer period of follow-up. However, because the mean duration of follow-up in our study was already relatively long, 64.42 months, it does not seem that a major portion of these ATM patients would have been diagnosed with MS with still longer periods of follow-up. Lastly, myelin-oligodendrocyte glycoprotein (MOG) antibody has recently been found in patients with demyelinating diseases of the CNS, including LETM. We speculate that at least some of our patients with ATM are associated with MOG antibody [33]. Further study is needed to determine the exact cause of this relatively high incidence of ATM among Koreans.

The current criteria for definite NMO [2] and the clinical features of NMOSD [3] seem to be useful in screening for NMO, because most of our patients in NMO groups met these criteria and/or had those clinical features. However, in our cohort, the criteria still seem to have some limitations, as follows: 1) Fifty patients with LETM (25% of our demyelinating cohort) and thirteen patients with bilateral simultaneous or recurrent optic neuritis (7% of our demyelinating cohort) did not have AQP4-Ab. This showed that the clinical features of NMOSD are not specific for NMO, at least in Asians. 2) A small number of patients (n = 2, 5.4% of our NMO group), who did not show these clinical features, still showed positive AQP4-Ab assay results (Figure 1). This result is in accord with recent studies evaluating the diagnostic utility of these clinical features [34,35]. Thus, despite the current diagnostic criteria and clinical features of NMOSD [2,3] seeming to be useful, they still appear to require further revisions, especially if they will be applied to Asians.

Our study has several limitations. First, it was not a population-based study. However, it involved a relatively large number of consecutive patients from three major hospitals in Korea, in the assessment of AQP4-Ab positivity and the ratio of NMO to MS in Korean patients with IIDDs. Second, some patients with AQP4-Ab can manifest isolated brain lesions in the early stages of the disease [36], which was not included in our list of clinical features of NMOSD. This is because we have adapted the published diagnostic criteria or clinical features of NMOSD [2,3]. However, some patients with isolated cerebral involvement and AQP4-Ab can show the same clinical course and/or response to treatment as do those with definite NMO [37].

## Conclusions

The incidence of NMO may be similar to that of MS in Korean patients with IIDDs. The AQP4-Ab positivity was 18.2% in our multi-center cohort of Korean patients with IIDDs, which is much lower than that reported previously from single-center studies in Asia (33–39%) [10,37]. ATM not associated with NMO or MS could be found in more than 40% of our patients with IIDDs, which mainly seems to be due to the low prevalence of MS in Korea. Additional population-based studies with AQP4-Ab testing are needed to determine the exact incidence of NMO, as well as the other diverse types of IIDDs, in Korea.

## Competing interests

Dr. Waters is a named inventor on a patent relating to assays for the detection of antibodies to Lgi1, Caspr2, and Contactin2, and may receive royalties for this technology. Dr. Waters receives research support from the Oxford NIHR Biomedical Research Centre and has received a speaker honorarium from Biogen Idec, Japan.

Dr. Kim Sung-Min, Dr. Woodhall, Dr. Kim Jee-Eun, Dr. Yang Ji Won, Dr. Yang Hyeran, Dr. Sung Jung-Joon, Dr. Park Kyung Seok, and Dr. Lee Kwang-Woo declare that they have no competing interests.

## Authors’ contributions

SMK, PW, and KSP conceived of the study, participated in its design and coordination, and helped to draft the manuscript. MK performed the AQP4-Ab assay. JWY, HY, JE, JJS, and KWL participated in the data collection. All authors read and approved the final manuscript.

## Pre-publication history

The pre-publication history for this paper can be accessed here:

http://www.biomedcentral.com/1471-2377/14/93/prepub
